# Bloody Aggressive: A Case Report on a Fungating Breast Mass in a Paraplegic Requiring Emergency Mastectomy

**DOI:** 10.7759/cureus.10952

**Published:** 2020-10-15

**Authors:** Asad Ullah Sabir, Sarah Sabir, Sundas Abbas

**Affiliations:** 1 Emergency Medicine, Rawalpindi Medical University, Rawalpindi, PAK; 2 Internal Medicine, Rawalpindi Medical University, Rawalpindi, PAK; 3 Dermatology, Rawalpindi Medical University, Rawalpindi, PAK

**Keywords:** fungating breast mass, emergency mastectomy, hemorrhage, infiltrating ductal carcinoma

## Abstract

Fungating malignant lesions pose a huge deal of agony to the patients. Their management is also deemed as difficult by most physicians. This report describes a case of a 45-year-old paraplegic female with delayed presentation of a very aggressive fungating left breast mass, which was diagnosed as invasive ductal carcinoma. The uncontrollable hemorrhage had the surgeons succumb to the option of emergency mastectomy as a palliative treatment to save the life of the patient. Hence, we infer that the emergency mastectomy in a hemorrhagic fungating breast lesion can be life-saving and can be performed with little to no risk to the patient. Such a procedure, surprisingly, has never been documented in the surgical literature before.

## Introduction

Breast cancer is a common misfortune experienced by women across the globe, and Pakistan has a higher rate than any country in Asia [1]. One out of every nine women suffers from this cancer at any time in their lives [2]. Added to this, it is generally diagnosed at more advanced stages than in other countries, owing to the poor socioeconomic status of the women in this country [3].

Locally advanced breast cancer, such as fungating breast cancer, is a category of breast cancers that usually present with large and very advanced tumors, regional lymphadenopathy, and direct extension of the tumor to the chest wall and/or skin, in the absence of distant metastasis [4]. Societal and cultural barriers, lack of awareness about their malignant nature, and economic restraints are the factors that contribute towards the late presentation of breast lumps in developing countries. Often women seek medical attention when the lump has grown to a very large size, and/or has developed a fungating mass [5].

Fungating malignant lesions have ulcerative and necrotic characteristics and manifest with pain, disfigurement, hemorrhage, odor, and infections [6]. Fungating masses are a nuisance that not only have a psychosocial impact but are also associated with high rates of mortality. Patients with fungating lesions tend to shy away from medical treatment in the fear of embarrassment. Management of such cancers is particularly difficult and is aimed at improving the quality of life, as the disease is very aggressive and incurable. Palliative treatment options such as radiation, wound cleaning, debridement, cleaning, and topical analgesia are commonly adopted. Embolization and cauterization to control bleeding may be done. Chemotherapy is usually done to downstage the tumor [7]. Surgical resections are also common.

Surprisingly, the literature rarely mentions the use of urgent mastectomy as a potential modality of palliative care for fungating breast masses that may be fatal if left to the mercy of other treatments that are slow in their effects compared to resection, and not usually successful.

## Case presentation

**Figure 1:**
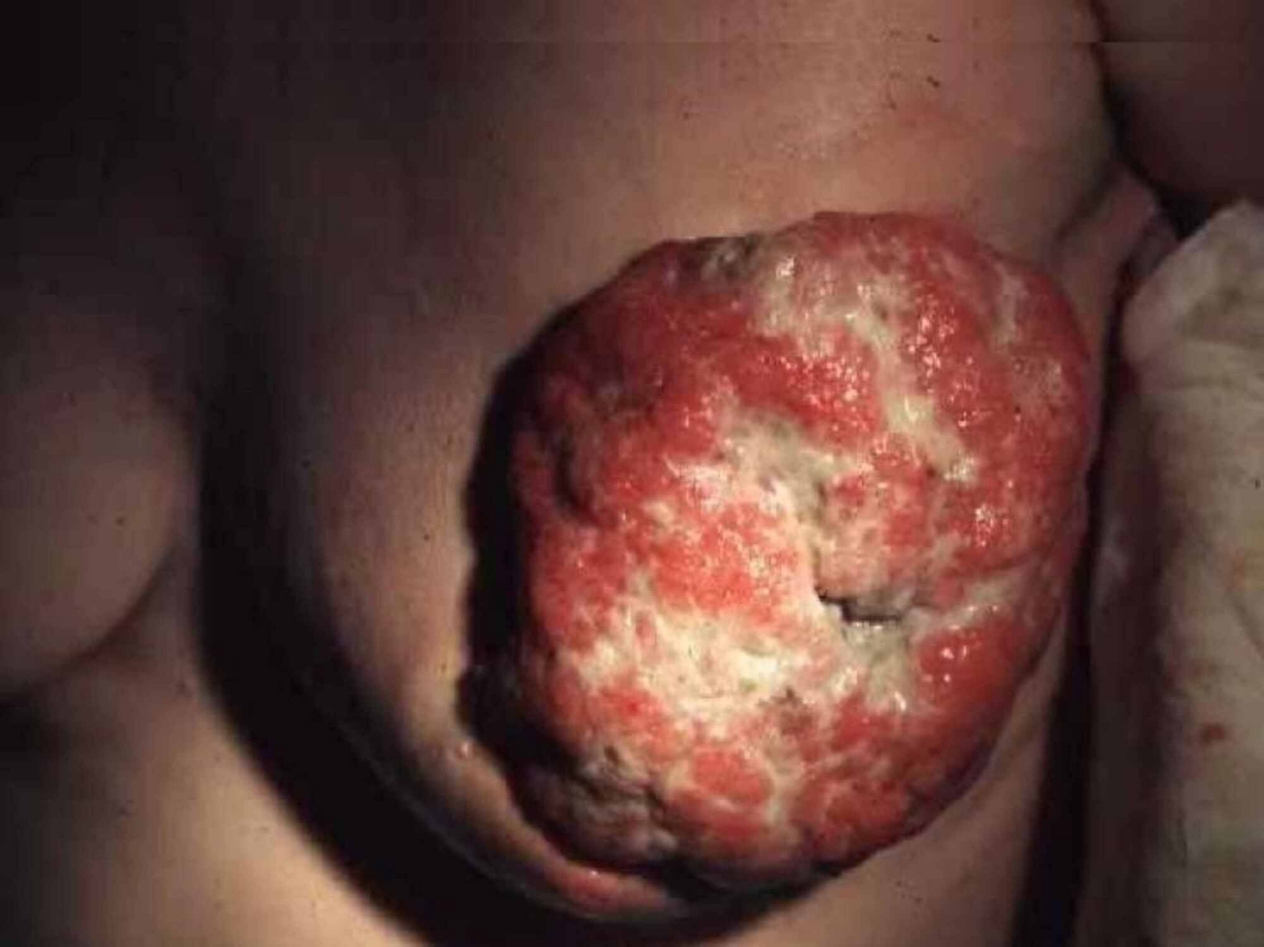
Fungating mass in the left breast in the 45-year-old female

A 45-year-old nulliparous, paraplegic patient from a remote rural area of Azad Kashmir, Pakistan, presented to the emergency department at Holy Family Hospital, Rawalpindi, with a fungating breast mass in the superior quadrants of her left breast. Her past medical history was significant for a fall from a mountain 20 years ago that rendered her paraplegic. She was in her usual state of health almost six months ago, when she felt a mild tearing pain in her left breast. Palpation by the caretaker revealed a lemon sized round mass. The surface of the breast and nipple were unremarkable. The mass continued to grow in size over the next four months, reaching the dimensions of an orange, and eroding through the upper outer quadrant of the breast. The patient’s attendant used to squeeze the breast to evacuate the ‘wound’- and reported periodic removal of ‘blood and flesh’ for the next one month. The ulcerative and the fungating mass progressively increased in size and protrusion, and the bleeding started to occur spontaneously once a day, of about 50 ml in quantity. This continued for a week after which she finally sought medical attention. On presentation, her blood pressure was 115/95 mmHg, pulse rate was 110/min, and respiratory rate was 19/min. Examination revealed a 15x15cm lump in the upper outer quadrant with a 5x7cm area of ulceration involving the nipple-areola complex as seen in Figure 1, with a fixed fungating mass with profuse bleeding and foul odor. Left axillary lymphadenopathy was present. There were no systemic symptoms. Her body mass index was 20 kg/m2 and she had lost 25 lb in four months. Family history was insignificant for any cancer, and she never smoked or drank alcohol. She reported regular five-day menstrual cycles and her age of menarche was 13. Wound debridement was done and a Tru-cut biopsy was performed. The sample was sent to the laboratory and the wound was dressed and bandaged. Systemic examination was unremarkable. A CT scan was done which revealed a huge mass in the central portion of the left breast adjacent to the anterior chest wall as well as metastatic pulmonary nodules in both lungs. The bone scan was fortunately negative for bony metastasis.

The histopathology report diagnosed it as infiltrating ductal carcinoma grade III and the tumor was not estrogen receptor and progesterone receptor (ER/PR)/Her-2neu receptive. A meeting was called, and it was decided to use antibiotics, analgesics, blood transfusions, and chemotherapeutic drugs until the mass could be reduced in size and resected. The wound was regularly cleaned and dressed every day. Her first round of chemotherapy was scheduled seven days later. Unfortunately, only five days later, upon dressing of the wound, the fungating mass started bleeding uncontrollably. The patient developed instant hemodynamic instability. Fluid resuscitation was done but to no success and she had to be taken to the operation room for an emergency mastectomy to save her life.

The procedure conventionally known as ‘Toilet Mastectomy’ was done. Resection of the breast was done followed by axillary clearance. Hemostasis was secured and a drain was placed. The fat was closed with Vicryl 2.0 and the skin was closed with a stapler. The specimen was sent to the laboratory for histopathology.

The patient was monitored for the next five days, and two pints of blood were transfused to normalize the hemoglobin level which was down to 6.1 g/dl. She reported immediate relief of pain and was extremely grateful to the surgeons. She was subsequently discharged and was advised to follow up regularly for chemotherapy and radiotherapy sessions. The follow-up results, however, were not available at the time of writing the report.

## Discussion

Although Pakistan has the highest rate of breast cancers in the whole of the continent, the government only spends 0.57% of its gross domestic product in the health sector. In addition, the awareness of mammography and practice of self-breast examination (SBE) as screening measures for breast cancer is also very sparse [8]. This stems from the fact that emphasis is not placed on women’s education in our society and the statistics of breast cancer are not given enough significance. A survey conducted in Karachi, Pakistan, in 2007 showed that about 20% of women did not even believe breast cancer occurs in Pakistan, and only 30% believed that it is a fatal disease [9]. Most of the working doctors in the community are males, and the cultural norms do not sanction clinical breast examinations by male primary physicians, which is why breast screening goes unchecked even in women who seek medical attention. Research showed that only 24% of the male general physicians performed clinical breast examination [10].

In developing countries, ductal carcinoma in situ accounts for only 0.6% of all reported cancers. The rest of the 99.4% are invasive carcinomas as opposed to invasive cancers in the developed world, which amounts to only 80% [10]. This can perhaps be attributed to the genetic factors as well as late presentation as regular screening is not giving priority. The downside to this is that the prognosis worsens as cancer progresses with time, therefore women with breast cancers usually have a high rate of morbidity and mortality in our community. The doctors exhaust their options trying to downgrade the tumor and as in our case, had to operate via emergency toilet mastectomy. Astonishingly, such a treatment is often observed in many tertiary care hospitals of Pakistan, although it has not been documented abroad.

A similar case of a bleeding fungating breast mass was reported in the literature for which the doctors proceeded with transcatheter arterial embolization (TAE) instead of surgery. They used the prior CT scan to guide the embolization using 300 to 500 μm and 500 to 700 μm microspheres with a 4-French angled hydrophilic catheter. The left internal mammary artery was fully blocked. The hemorrhage was successfully controlled [11]. However, in a developing country like Pakistan, interventional radiology services are not easily available and accessible to everyone so there is no option left but surgery.

A sad reality is that the handicapped people in our community are not very well looked after. They are left at the mercy of their family members to decide their quality of personal hygiene and medical care. As we saw in our case, compressions were regularly given to drain out the wound, which possibly increased the hemorrhagic propensity of the tumor and increased the chances of infection.

## Conclusions

Due to the financial constraints, mammography is not widely available, accessible, or affordable for most women, therefore, a system providing regular clinical breast examinations to every woman by trained workers should be approved. In developing countries where breast cancers usually present late, emergency mastectomy should be considered as a palliative surgical procedure for aggressive bleeding tumors. Evidence-based guidelines should be constructed for increased success rates.
